# Horizontal Bilayer for Electrical and Optical Recordings

**DOI:** 10.3390/ma5122705

**Published:** 2012-12-10

**Authors:** Philipp Bartsch, Claudius Walter, Philipp Selenschik, Alf Honigmann, Richard Wagner

**Affiliations:** 1 Biophysics, Department of Biology/Chemistry, University Osnabrueck, Barbarastr. 13, Osnabrueck 49076, Germany; E-Mails: philipp.bartsch@biologie.uni-osnabrueck.de (P.B.); claudius.walter@biologie.uni-osnabrueck.de (C.W.); philipp.selenschik@biologie.uni-osnabrueck.de (P.S.); ahonigm@gwdg.de (A.H.); 2 Ionovation GmbH, Westerbreite 7, Osnabrueck 49084, Germany

**Keywords:** horizontal bilayer, simultaneous electrical-optical recordings, membrane dynamics, lipid diffusion, supported bilayer

## Abstract

Artificial bilayer containing reconstituted ion channels, transporters and pumps serve as a well-defined model system for electrophysiological investigations of membrane protein structure–function relationship. Appropriately constructed microchips containing horizontally oriented bilayers with easy solution access to both sides provide, in addition, the possibility to investigate these model bilayer membranes and the membrane proteins therein with high resolution fluorescence techniques up to the single-molecule level. Here, we describe a bilayer microchip system in which long-term stable horizontal free-standing and hydrogel-supported bilayers can be formed and demonstrate its prospects particularly for single-molecule fluorescence spectroscopy and high resolution fluorescence microscopy in probing the physicochemical properties like phase behavior of the bilayer-forming lipids, as well as in functional studies of membrane proteins.

## 1. Introduction

Model membrane systems have proven to be useful tools for probing the molecular properties of lipid bilayers and transmembrane proteins. Two basically different planar model membrane systems evolved:

i Supported bilayers spread on the surface of various supporting substrates like glass, mica or Si/SiO_2_.
ii Free-standing bilayer which is spread across a micro-aperture of 30–200 µm within a thin (≈12.5–50 µm) PTFE-septum or other types of suited polymer films.

A variety of different systems for creating and exploiting artificial lipid bilayers or lipid membrane biosensors in the nanoscale and microscale regime have been developed recently [1,2]. Here, we describe a universal usable microchip that allows formation of horizontal free standing and supported bilayer with long lifetime for combined or simultaneous electrical and high resolution fluorescence recordings. We first briefly summarize and emphasize basic critical topics for setting up such a system. Similar combined or simultaneous optical recordings on channel proteins have been performed previously in artificial bilayers [3,4], giant liposomes [5,6] oocytes [7,8,9] and the droplet interface [10]. Further on, we describe a combination of the above two approaches using cushions of natural or synthetic hydrogels as bilayer support which up to now have not been used frequently, despite offering significant advantages in many aspects [11,12,13,14].

For the measurement of channel and transporter mediated membrane translocation of ions and non-charged solutes, free-standing bilayers are superior to surface spread bilayers for several reasons, the main of which is the well-defined dielectric isolation of two water phases by the model membrane (GΩ-seal) enabling, for example, electrical recording of single ion channel currents.

Free-standing artificial bilayers are mostly fabricated by the so called “painted-bilayer” technique [15]. Bilayers are “painted” onto micropores (d ≈ 30–200 µm) within a Teflon^®^ septum and the development of a bilayer is determined by measurement of the characteristic bilayer capacitance [16]. These model bilayer systems can be used to study molecular properties of single ion channels in a chemically well-defined environment. Supported bilayers on mica, glass, and Si/SiO_2_ substrates have been used for studies of biomolecules using optical and scanning probe microscopy [7,8,9]. To form bilayers on solid substrates, various spreading techniques where protein-containing vesicles are directly spread onto a hydrophilic surface were used. This technique has been mainly used in combination with optical probes which allow for obtaining information on the physico-chemical properties of the bilayer membrane [17,18].

Here, we describe construction details, required materials, and the procedure to set up a horizontal bilayer microchip system with long-term stable bilayers which optionally can be stabilized by cushions of natural or synthetic hydrogels as bilayer support. We demonstrate the prospects of this microchip particularly for single-molecule fluorescence spectroscopy and high resolution fluorescence microscopy. We will present data on single-molecule tracking of fluorescent labeled lipids and combined opto-electrical recordings to monitor the functional membrane integration of the toxic porin PorB from *Neisseria gonorrhoeae*.

## 2. Results and Discussion

### 2.1. Microchip Setup—Geometric Features, Materials of the Chip, Fabrication of the Aperture Hole & Chip Assembly

**Figure 1:**
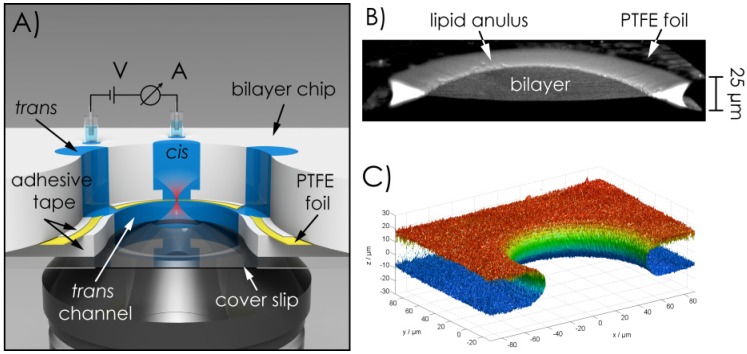
(**A**) Principal layout of the bilayer microchip construction; (**B**) Cut-through of a 3D-scan of a bilayer; (**C**) Isosurface modeling of a 3D-scan of an aperture-hole.

The geometric features of the chip are outlined in Figure 1. The different layer components of the chip were assembled in sandwich architecture (Figure 1A) to meet the requirements of the electrical recordings and the optimal distance of the bilayer within the optical path (see experimental section for details). The most critical component within the sandwich architecture of the bilayer chip is the material of the septum/foil and the characteristics of the aperture hole which serves as bilayer scaffold. Based on long term observations in our lab (>10 years [19,20]) we had to realize that for the fabrication, handling and long-term stability (>2 h) of the bilayers, the ratio of the diameter of the hole and the thickness of the supporting foil (aspect-ratio, ar) should not exceed a critical value of ~8 (ar ≤ 8). With respect to lipid annulus determined bilayer stability an aspect ratio of ar = 1 would be ideal, although, in practice, difficult to achieve [21]. Secondly, the inner surface and the border surface of the hole should be perfectly smooth. Finally the material of the bilayer support foil should provide dielectric insulation combined with a matched hydrophobicity. PTFE is lipophilic enough to be sufficiently wetted by *n*-decane (or many other alkanes) which, in turn, provides the support for the amphiphilic lipids which accumulate at the *n*-decane/air resp. *n*-decane/buffer-boundary. For these characteristics and their mechanical stability, fluor-polymer films of different compositions have been shown to be the best choice (see experimental section for details).

We use a self-constructed electro-mechanical device (electro-mechanical-micro-hole-generator, EMMHG) which allows generating aperture-holes in plastic films with well-defined diameter and extremely smooth surface in a very reproducible manner (details are given in the experimental Section). Figure 1C shows an isosurface modeling of a 3D-scan of an aperture-hole which perfectly meets all of the above described requirements. Bilayer microchips which comply with the above requirements are also commercially available (Ionovation GmbH, Osnabrueck, Germany).

### 2.2. Bilayer Fabrication and Stability

#### 2.2.1. Painting and Other Techniques

**Figure 2:**
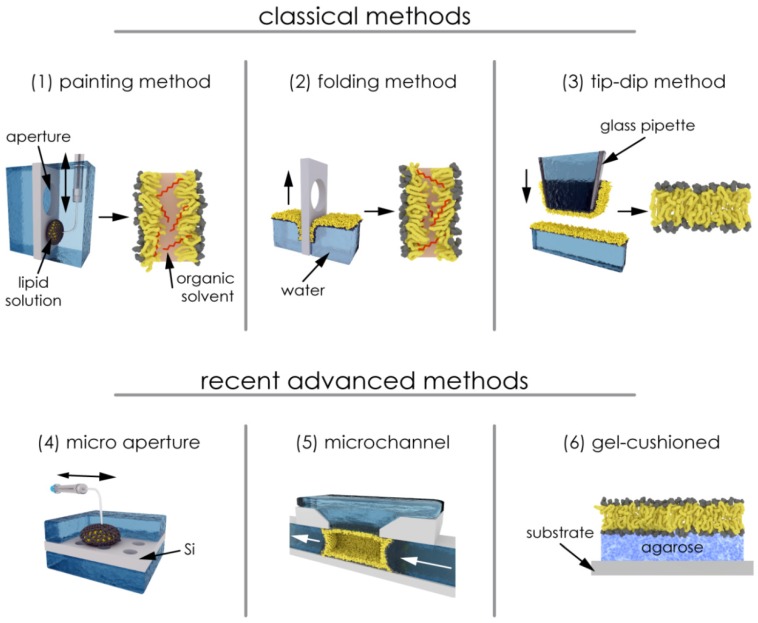
Schematic illustration of the conventional and micro-fabricated methods for the preparation of artificial bilayer membranes. (1) Painting method (black membrane); (2) folding method (reduced-solvent BLM); (3) tip-dip method (solvent-free BLM); BLM formation in (4) micro-fabricated aperture; (5) micro-channel and (6) BLM cushioned on agarose gel (see also [26]).

Conventional planar bilayer membranes have been formed by classical painting technique (black lipid membrane (BLM)) [22], monolayer folding [23], and tip-dip methods [24], (Figure 2, upper row). These membranes have been widely used for functional analysis of channel-forming proteins. Recent advances use micro-fabricated devices with mainly horizontally oriented planar bilayers formed across sub-micrometer to ~200 µm apertures [25,26] (Figure 2, lower row). These setups are also suitable for combined optical and electrical readout.

#### 2.2.2. Solvent and Residual Solvent: Effects on the Bilayer Physical State

For fabrication of long-term stable artificial lipid membranes, the use of hydrocarbon solvents is essential, because the bilayer is thinned out from a torus of a solvent-lipid mixture deposited across the hole of the supporting septum. Similarly, the practicable version of the folding method [27] (see Figure 2) is also based on the deposition of lipid-solutions on the water surface of both compartments. Therefore, the solvent may also partition inside the actual bilayer, thereby causing changes in the physico-chemical properties of the membrane.

We have previously addressed this question and our results showed that the lateral and rotational diffusion constants of lipids depend on the nature of the hydrocarbon solvents used for bilayer preparation. However, our measurements on phase separation in ternary lipid bilayers showed that only trace amounts of short chain hydrocarbons like hexane remain in the bilayer. Moreover, they do not change the basic physico-chemical properties of the bilayer membrane except for a more fluid membrane and a depressed transition temperature (*T*_m_) as compared to solvent-free bilayers [28]. Solvent partitioning was also found to increase the bilayer thickness, probably by intercalation of the solvent molecules between the inner and outer leaflets [27,29,30]. Generally, long chain hydrocarbon solvents should be preferred over short chain solvents, because the partitioning in the membrane decreases with increasing chain length (decane, hexadecane, squalene) [28].

Lipid composition of the artificial bilayer membranes determines their physical properties and stability as well as the function of integral membrane proteins [31]. Lipid extracts from biological membranes as well as a large variety of synthetic lipids have been used for the generation of stable bilayer membranes [2]. In general, it should be considered to use lipids with *T*_m_ below room temperature, *i.e.*, lipids which are in a fluid state at room temperature. Due to the large number and variety of lipids compositions used [2,31], we refer only to a few examples of lipid compositions typically employed in artificial bilayers (see Table 1).

**Table 1:** Lipid Extracts and synthetic lipids used for artificial lipid bilayers.

Name	Composition	Type	References
Azolectin (Type IV-S, ≥30%)	PC (40%), PE (33%), PI (14%), lyso-PC (5%), CA (4%)	extract	[32]
*E. coli* Total Lipid Extract	PE (57,5%), unknown (17,6%), PG (15,1%), CA (9,8%)	extract	Avanti Polar Lipids, Inc. (Alabaster, AL, USA)
*E. coli* Polar Lipid Extract	PE (67%), PG (23,2%), CA (9,8%)		[14]
	DOPC:DOPE (70%:30%)	synthetic	[33]
	DOPC:DMPC:CO (1:3:1)	synthetic	
	DOPC:DSPC:CO (2–1:1:1)	synthetic	[28]
Outer mitochondrial membrane	PC (51%), PE (35%), PI (9%), CA (4%), PS (1%)	synthetic	[34]
Inner mitochondrial membrane	PC (42%), PE (38%), CA (17%), PI (2%), PS (1%)	synthetic	[34,35]

**Abbreviations:** CA: cardiolipin; CO: cholesterol; DMPC: 1,2-dimyristoyl-*sn*-glycero-3-phosphocholine; DOPC: 2-dioleoyl-*sn*-glycero-3-phosphocholine; DSPC: distereoyl-*sn*-glycerol-3-phosphocholine; PC: phosphatidylcholine; PE: phosphatidylethanolamine; PG: phosphatidylglycerol; PI: phosphatidylinositol; PS: phosphatidylserine.

#### 2.2.3. Contaminations Impeding Electrical and Optical Single-Molecule Measurements

**Figure 3:**
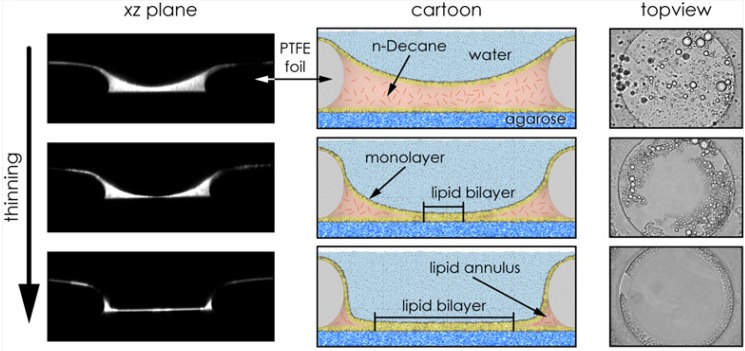
The left and right column shows an emerging agarose-supported horizontal bilayer made of azolectin type IV-S in *xz* and *xy* plane, respectively (see experimental section for details). The lower left Figure shows the bilayer deposited by hydrostatic pressure directly on the agarose support. The lipid is thinned out top–down, as indicated by the arrow. The middle column illustrates this process.

Using lipid extracts from natural membranes for artificial bilayer fabrication requires special precautions for both electrical and fluorescence-optical recordings. These extracts, even when purified with large effort, in many instances still contain minute traces of peptides or partially degraded proteins which either form membrane pores or destabilize the membrane in a manner to produce gating like current fluctuations in response to applied membrane voltages in the typical voltage clamp configuration [19,20]. Actually, almost all of the commercially available lipid extracts from natural membranes, particularly from plant membranes, contain residual traces of chromophores which produce an extensive fluorescence background in a high-resolution fluorescence setup (see Figure 3). Even a mole fraction of 1 ppm of a contaminant fluorophore means that there is nearly one unwanted dye molecule in a diffraction limited spot even at 488 nm. From this, it is evident that mixtures of high purity synthetic lipids are superior to lipid extracts, although they are usually much more expensive. Despite that, according to our long-term experience, lipid extracts from natural membranes in combination with careful controls provide an economical basis for the highly reproducible fabrication of long-term stable artificial horizontal bilayers for electrical recordings [19].

#### 2.2.4. Polymer-Supported Bilayer

Lipid membranes that are physically coupled to a polymer cushion represent a reasonable compromise between free-standing bilayers, which are thought to provide undisturbed membrane fluidity, and lipid-bilayer membranes adsorbed on solid substrates, which are assumed to largely reduce the natural fluidity of the bilayer membrane. Here, we briefly describe a variation of the polymer cushion method which starts with a fabricated horizontal free-standing bilayer where the lower compartment contained soluble polymers forming a hydrogel that provides a large ionic reservoir. This type of bilayer exhibits nearly the membrane fluidity of a free-standing bilayer; however, its overall stability is significantly increased. Figure 3 shows a confocal fluorescence scan of the *xz* plane and wide field *xy* plane view from a horizontal bilayer with an agarose cushion support below the membrane.

### 2.3. Bilayer Optical Recording

#### 2.3.1. Single-Molecule Fluorescence Recordings from Lipid Bilayer Membranes

Single-molecule fluorescence recordings from lipid bilayer membranes were conducted using the analysis of fluorescence intensity fluctuations of single molecules in a confocal volume. In particular, we applied the time-correlated single photon counting (TCSPC) confocal techniques FCS, FIDA and FLT [36]. Next, we used single-molecule wide-field imaging and fitting of a two-dimensional Gaussian to the observed two-dimensional intensity distribution of the point spread function (PSF) to determine the position and movements of the fluorescent labeled lipids and proteins [13,37].

**Figure 4:**
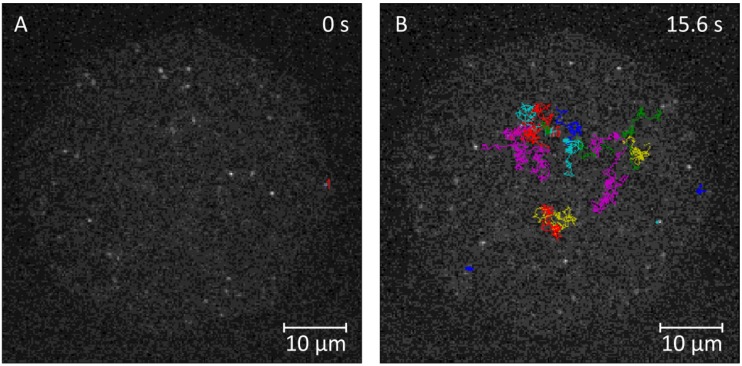
Glass-supported bilayer. (**A**) Representative trajectories of DPPE647N obtained from a glass-supported lipid bilayer at *t* = 0 s and (**B**) *t* = 15.6 s. Two modes of motion are evident in (**B**): Trapped/immobile (e.g., the blue spot, bottom left) and freely diffusing molecules (colored lines). Lag time (*t*_lag_) between two consecutive frames: 15.6 ms.

To verify fluidity properties and the stability of free-standing, hydrogel-cushioned and glass-supported bilayers, we studied the diffusion of labeled lipids (Atto647N-labeled DPPE) in these model systems using single-molecule fluorescence tracking (SMT) and confocal fluorescence–fluctuation spectroscopy. Experiments on free-standing and polymer-supported membranes were conducted with ternary lipid mixtures consisting of DOPC:DMPC:CO typically at a molar ratio of 1:3:1. Solid (glass)-supported bilayer membranes were generated by spreading small unilamellar vesicles (SUVs) made from DPPE647N:DOPC (molar ratio 1:1.3 × 10^9^) onto a cleaned (piranha etch) cover slip. Representative trajectories obtained from this model system are depicted in Figure 4.

The data were evaluated as follows: For every trajectory and lag time, a list of mean squared displacements (MSDs) was generated by averaging over all pairs of localized points:

(1) $$\langle \text{∆}r²(t_{\text{lag}})\rangle =\;\frac{1}{N_{\text{A}}}∙\overset{N_{\text{A}}-1}{\underset{i=0}{\sum }}[\vec{P}(i+t_{\text{lag}})-\vec{P}(i)]²$$

*N_A_* denoting the number of overlapping segments of length n and $\vec{P}$ the actual position of the molecule(s). The MSDs can subsequently be pooled and for every lag time, the cumulative distribution function (CDF) would be constructed as described by Schütz *et al.* [38]:

(2) $$P\left(r²,t\right)=1-\;e^{-\frac{r²}{4Dt}}$$

As predicted [39,40], the distribution of calculated diffusion coefficients is characterized by a gamma distribution. Equation 2 is readily expanded to account for fractions of two differently mobile components [38]:

(3) $$P\left(r²,t\right)=1-\left(\alpha ∙e^{\left(-\frac{r²}{r_{1}^{2}}\right)}+(1-\alpha )∙e^{\left(-\frac{r²}{r_{2}^{2}}\right)}\right)$$

**Figure 5:**
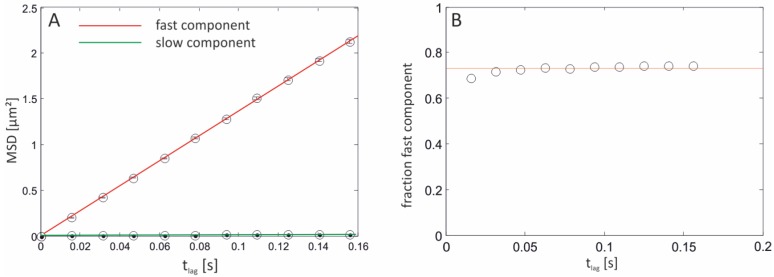
MSDs from a glass-supported bilayer. (**A**) Dependence of the MSD on the time lag set to 15.6 ms for individual molecules tracked over ≥50 frame numbers. A linear dependence of the MSD for the fast fraction on time is indicative of free diffusion. The regression line for the low-mobility component is almost asymptotic but reveals a marginal slope when magnified (not shown). $\bar{D}$ for the fast and slow fraction equates 3.42 µm^2^/s ± 0.014 µm^2^/s and 0.028 µm^2^/s ± 0.004 µm^2^/s, respectively. (**B**) The fraction of the fast component was determined to 73% ± 2% and is constant covering all time lags. Error bars are equal to $r_{i}^{2}/\sqrt{N}$, *N* denoting the total number of data points.

While α corresponds to the fraction of the fast moving particle, $r_{1}^{2}$ equates the mean square displacement of the fast and $r_{2}^{2}$ the mean square displacement of the slow component. However, it is difficult to discriminate between the one- or two-component models solely based on how well they fit. We therefore determined the maximum likelihood estimator(s) (MLE) of equations 2/3 and utilized a likelihood ratio test (LRT) to discriminate between the two nested regression models at a significance level of 5%. The application of this approach to data derived from glass-supported membranes is shown in Figure 5.

Here, the data were evaluated using a two-component MLE-model, yielding values of $\bar{D_{fast}}$ = 3.42 µm^2^/s ± 0.014 µm^2^/s for the high- and $\bar{D_{slow}}$ = 0.028 µm^2^/s ± 0.004 µm^2^/s for the low-mobility component with a value of 100% for two detected fractions. That is, at every time lag the LRT found the two-component model to be statistically significantly better than the one-component fit, proving the existence of at least two species of differently fast diffusing molecules. The determined value for the high-mobility component is in good agreement with the published lateral diffusion coefficient for DOPC membranes deposited on oxidized silicon (approximately 3.5 µm^2^/s at 23 °C [41]). Additionally, it agrees well with the value determined by Kalb *et al.* [42] (3.6 µm^2^/s ± 0.5 µm^2^/s) and is comparable since all experiments were conducted at room temperature and POPC, as well as the lipid used in this approach, DOPC, both have transition temperatures far below 23 °C. Ladha *et al.* [43] have shown that the second unsaturated alkyl chain present in DOPC does not impact the diffusion coefficient compared to POPC membranes. The low-mobility component is assumed to resemble the immobile fraction observed in fluorescence recovery after photobleaching (FRAP) measurements. The fraction for the fast component amounts 73% ± 2% and remains constant over all time lags the experiment relied on (Figure 5B). This value agrees well with data obtained for the recovery rate applying FRAP measurements (80%) on glass-supported POPC membranes [42]. It is possible, yet not quantified, that the solid-supported lipid bilayer is not homogeneously distributed over the entire oxide surface. These membrane defects in turn could alter the diffusion of lipids present at the edge of the bilayer or form microstructures giving rise to trapping events.

**Figure 6:**
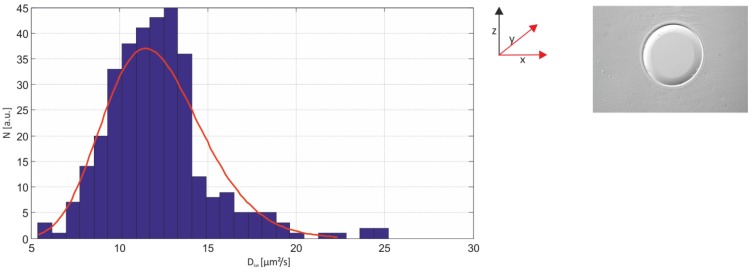
Cumulative histogram of diffusion coefficients for all tracked molecules diffusing in a free-standing lipid bilayer. The distribution is characterized by a mean of 12.2 µm^2^/s and a variance σ² = 4.3 µm^4^/s^2^. Top right: *xy* view of a horizontal lipid bilayer. Note the presence of the Plateau–Gibbs border. Bin sizes are equal to the square root of the number of data points generating the histograms.

**Figure 7:**
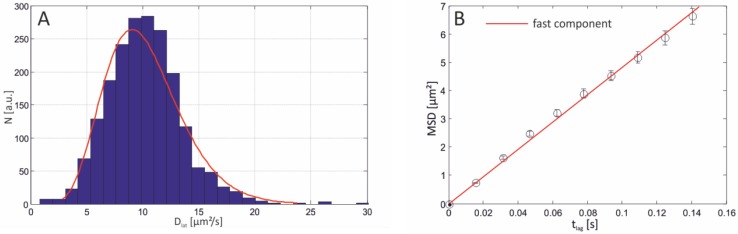
(**A**) Distribution of diffusion coefficients for 250 trajectories obtained from an agarose-supported lipid bilayer. The histogram is adequately fitted with the one-component model, characterized by a mean diffusion coefficient of 10.22 µm^2^/s and a variance of 3.461 µm^4^/s^2^. (**B**) Corresponding MSD *vs.* *t*_lag_ plot, revealing a linear dependence of the MSD on time and a slope of $\bar{D}=$ 10.013 µm^2^/s ± 0.298 µm^2^/s.

Figure 6 depicts the distribution of diffusion coefficients for measurements performed on black lipid membranes without any support. The annulus, a characteristic feature of artificial black lipid bilayers in general is clearly visible. The data of every single trajectory were fitted according to the one-component MLE model and a cumulative histogram of all determined diffusion coefficients corresponding to all molecules investigated was created. As predicted from theoretical analysis [40], the histogram follows a gamma distribution with a mean (μ=k∙θ) of $\bar{D}$ = 12.2 µm^2^/s and variance (σ² =k∙θ²)σ² = 4.3 µm^4^/s^2^ (Figure 7).

When performing single-molecule tracking experiments, the stability of the lipid bilayer is essential. Initial approaches to stabilize the lipid bilayer employed agarose as a hydrogel support [43,44] However, the agarose does not seem to significantly affect the lateral mobility of the Atto647N-labeled phosphor-(glycero)-(DPPE). The distribution is characterized by a mean diffusion coefficient of 10.22 µm^2^/s ± 1.86 µm^2^/s (Figure 7). This value is 2 µm^2^/s lower compared to measurements performed on free-standing membranes (Figure 6), indicating only a slight effect of the agarose support. Since the distribution is very well fitted by a one-component MLE fit, it is assumed that the observed motion relied on a single mobile class. Accordingly, the data were fitted with the one-component model, yielding a $\bar{D}=$ 10.013 µm^2^/s ± 0.298 µm^2^/s. Moreover, the mode of motion is suggested to be free (Figure 7B).

The variance of the fit does not allow for unambiguous discrimination between free-standing ($\bar{D}$ = 12.2 µm^2^/s ± 2.07 µm^2^/s) and agarose-supported ($\bar{D}$ = 10.22 µm^2^/s ± 1.86 µm^2^/s) model systems, suggesting that the agarose-supported membranes were in fact partially free-standing. This view is supported by FCS measurements (data not shown) on agarose-supported membranes which yielded a diffusion coefficient of 11.2 µm^2^/s, the latter value being in good agreement with those obtained from lipid bilayers without any support (see Figure 7).

**Figure 8:**
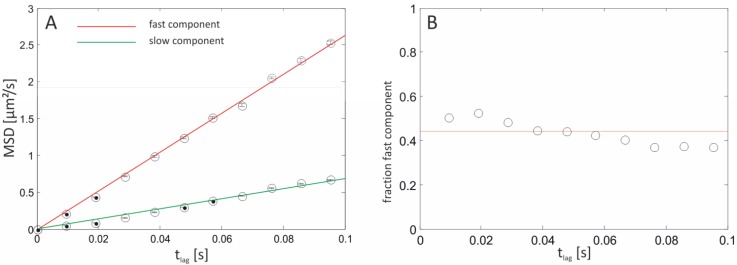
MSDs from a PLL-g-PEG supported bilayer. (**A**) Dependence of the MSD on the time lag set to 9.52 ms for individual molecules tracked over ≥50 frame numbers. Their MSDs are determined and pooled, yielding one value per time lag. A linear dependence of the MSD on time is indicative of free diffusion. Error bars are equal to $r_{\text{i}}^{2}/\sqrt{N}$, *N* denoting the total number of data. $\bar{D}$ for the fast and slow fraction equates 6.58 µm^2^/s ± 0.129 µm^2^/s and 1.718 µm^2^/s ± 0.091 µm^2^/s, respectively. (**B**) The fraction of the fast component amounted to 44% ± 8% and is constant covering all time lags.

**Figure 9:**
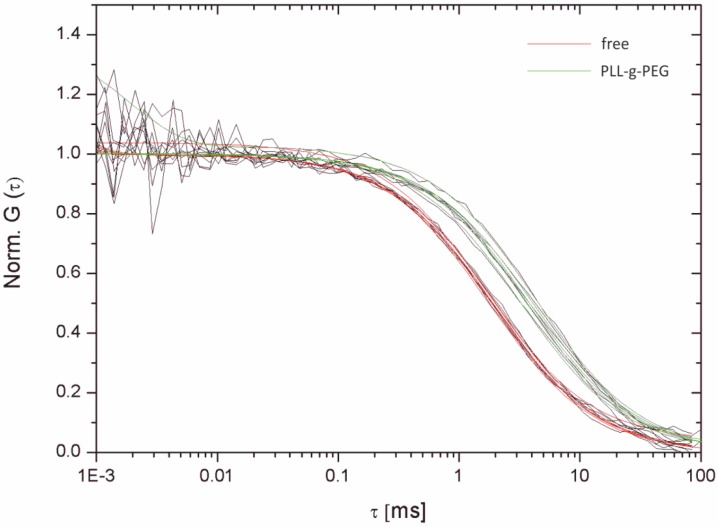
Autocorrelation curves for free-standing (red) and PLL-g-PEG-supported (green) lipid bilayer membranes. The mean diffusion times are clearly shifted towards longer values. The autocorrelation function is normalized to 1. $\tau_{\text{D}}$ values of 1.98 ms and 4.86 ms yielded diffusion constants of 13.51 µm^2^/s and 6.96 µm^2^/s for free-standing and PLL-g-PEG-supported lipid bilayers, respectively.

The distance between the lipid bilayer and the agarose polymer is a crucial point affecting the diffusional properties. However, it is questionable that the agarose polymer formed a smooth, homogeneous surface. Therefore, inhomogeneous distributed polymer-chain borderlines could be exposed to the bottom leaflet of the lipid bilayer, imposing fence-like constraints on the lateral mobility of the (labeled) lipids. At our conditions used (2.5% w/v agarose), the pore size at a setting temperature of 22 °C is approximately 100 nm [45]. Interestingly, a variation of the agarose concentration and thus the pore size (ranging from 0.5% to 3% (w/v)) had no effect on the obtained diffusion coefficients. Even if confinements in the range of 100–300 nm obstructed the mobility they would have remained unresolved at the applied frame-rates. When horizontal lipid bilayers were deposited on a poly(L-lysine)-g-poly(ethylene glycol) (PLL-g-PEG) cushion support the diffusion of lipids was split up into two components with ${\bar{D}}_{\text{fast}}$ = 6.58 µm^2^/s ± 0.129 µm^2^/s and ${\bar{D}}_{\text{slow}}$ = 1.72 µm^2^/s ± 0.091 µm^2^/s (Figure 8). Diffusion in PLL-g-PEG-supported lipid bilayers seems to be exclusively free since the MSD shows a linear dependence on time as is evident from Figure 9.

In order to further substantiate the SMT data additional FCS measurements on both freestanding and PLL-g-PEG-supported lipid bilayers were performed. The results are depicted in Figure 9. The autocorrelation curve is clearly shifted to longer times for PLL-g-PEG-supported lipid bilayers, indicating a decrease in mobility. Evaluation of the mean transition time of the particles $\tau_{D}$ yielded 1.98 ms and 4.86 ms, respectively. This corresponds to diffusion constants of 13.51 µm^2^/s and 6.96 µm^2^/s for freestanding and PLL-g-PEG-supported lipid bilayers at a known $\tau_{D}$ of the reference dye (Atto655 maleimide, *D* = 410 µm^2^/s). These values are rather in good agreement with those determined earlier applying SMT (12.2 µm^2^/s ± 2.07 µm^2^/s and 6.58 µm^2^/s ± 0.129 µm^2^/s for free-standing and PLL-g-PEG-supported lipid bilayers, see Figure 6 and Figure 8). This is presumably due to the different length scales the experiments were performed at. In particular, an area corresponding to π∙r²= 1256 µm^2^ is illuminated using SMT, *r* corresponding to the radius (20 µm) of the illuminated circular area. The subsequent analysis relied on the individual motion of the molecules, but is typically about a few pixels (at a 60× magnification, one pixel corresponds to 0.267 µm). FCS measurements are performed at even smaller length scales ($\omega_{0}=$ 0.326 µm, corresponding to a circular area of 0.33 µm^2^). Therefore, partial binding events, collisions or confinements are statistically more probable during SMT experiments, partially explaining the small discrepancy in the determined diffusion constants.

The diffusion coefficients obtained for PLL-g-PEG-supported lipid bilayer membranes are considerably higher than the values for glass-supported lipid bilayers (6.58 µm^2^/s compared to 3.42 µm^2^/s for the high- and 1.72 µm^2^/s as well as 0.028 µm^2^/s for the low-mobility component). Basically, two models exist that are applicable to explain this observation. At first, PLL-g-PEG is a poly-cationic polymer, because its positively charged primary protonated amine groups at pH 7.2 present at the poly(l-lysine) backbone. These interact electrostatically with the oxide layer of the glass coverslip, in this way partially shielding the negative charges from the lipid bilayer. The positively charged headgroups of the labeled lipids are still affected, although to a lesser extent. Therefore, the increase of the obtained values for the diffusion coefficients at least for the high-mobility component compared to the glass-supported membranes is comprehensible.

Secondly, the fast component observed for PLL-g-PEG supported lipid bilayers is thought to arise from the improved lubricating properties of the water induced by PLL-g-PEG [46]. The poly(l-lysine) backbone is linked to a variably long polymer consisting of ethylene glycol via an amide linkage. In our case, the polymer consists of 47 ethylene glycol monomers, corresponding to an approximate chain length of 10 nm. However, this is only true if the chain is linearly stretched. The chains could as well exhibit a purely random orientation which would in turn decrease the thickness of the layer. It is questionable if the polar headgroups of the (labeled) lipids are directly exposed to the PEG-chains or face the lubricating water layer. The suggested presence of at least two distinct components is partially attributed to frictional forces induced by the PEG molecules imposed on (labeled) lipids diffusing in the bottom leaflet of the lipid bilayer. This is equally applicable to the glass-supported system, where a hydration layer between support and lower leaflet is thought to impose frictional forces on the diffusing objects as suggested by Sonnleitner *et al.* [47].

We propose that, based on the coupling of both leaflets [48,49], altered diffusion in the bottom leaflet of PLL-g-PEG-supported bilayers is transferred to its upper counterpart, especially concerning the employed lipids of different chain lengths (18:1 for DOPC and 14:0 for DMPC, respectively). The strongly affected bottom leaflet ($\bar{D}=$ 1.718 µm^2^/s ± 0.091 µm^2^/s) would thus affect motion in the upper one ($\bar{D}=$ 6.58 µm^2^/s ± 0.129 µm^2^/s).The mean fraction of the fast component was 44% ± 8% and constant for all time lags (see Figure 8). Likewise, 56% ± 8% of all detected molecules belonged to the slow component, nicely matching the above model. However, it has been shown utilizing FCS measurements in combination with iodide fluorescence quenching [50] that the obtained diffusion coefficients in both the inner and outer leaflet of a (polymer) supported lipid bilayer are similar and thus relatively independent of their relative position to the support [41].

The values for the diffusion coefficients determined for the four different bilayer systems are summarized in Table 2.

**Table 2:** Summary of the determined diffusion coefficients for Atto647N-labeled DPPE in ternary lipid mixtures of DOPC:DMPC:CO at a molar ratio of 1:3:1 or pure DOPC in various model systems.

Bilayer system	*D*_fast_ (µm^2^/s)	*D*_slow_ (µm^2^/s)	Fraction_fast_
Free-standing	12.2 ± 2.07	–	1
Free-Standing (FCS)	13.51 ± 1.04		
Agarose-supported	10.22 ± 1.86	–	1
PLL-g-PEG-supported	6.58 ± 0.129	1.718 ± 0.091	0.44 ± 0.08
PLL-g-PEG-supported (FCS)	6.96 ± 1.1	–	1
Glass-supported	3.42 ± 0.014	0.028 ± 0.004	0.73 ± 0.02

#### 2.3.2. Fluorescence Anisotropy—Lipid Mixtures and Phases

Time-resolved (TRA) and steady state (SSA) fluorescence anisotropy is sensitive to the dynamics of lipid probes in the membrane [18]. In contrast to FCS, which senses long range lateral movements in the microsecond-to-millisecond range, fluorescence anisotropy is used to measure rotational dynamics (rotational correlation-time) and membrane order as sensed by the SSA. The rotational correlation times and the limiting and residual anisotropy of lipid probes probed by TRA/SSA report the short-range interactions within the very local environment of the studied probe, which can be related to the order of the acyl chains of lipids [51].

**Figure 10:**
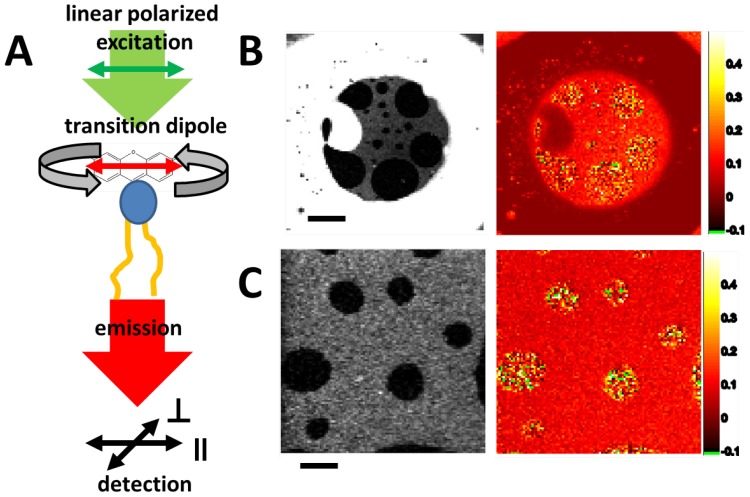
Steady state anisotropy of DPPE647N in ternary lipid bilayers. (**A**) Scheme for measuring lipid rotation with fluorescence anisotropy. Linear polarized light excites lipid-probes with parallel dipole orientation. During the excited state lifetime, the probe rotates. The emitted light is split according to parallel and perpendicular polarization and is detected simultaneously on both channels. The ratio of parallel to perpendicular detected photons is proportional to the rotational mobility of the probe. (**B**) Intensity and anisotropy image of a whole bilayer including the torus (scale bar 20 µm). The membrane was made of DSPC:DOPC:CO in a molar ratio of 2:2:1. (**C**) Zoom of the liquid-disordered (L_d_)/liquid-ordered (L_o_) phase distribution in the ternary bilayer (scale bar 5 µm). Pixel colored in green had too low an intensity (less than five counts per channel) to be evaluated for the anisotropy image.

In the intensity and anisotropy image of the whole bilayer, including the torus, the liquid ordered (L_o_) phase, is represented by the dark region in the intensity image (Figure 10B, left). The anisotropy in the torus (Figure 10B, right) was close to zero, indicating very low order and fast rotation due to the presence of the solvent. The liquid disordered (L_d_) phase in the bilayer corresponding to the bright region in the intensity image had a mean anisotropy of 0.17, indicating ordering and restriction of rotational movement of lipids due to bilayer formation. The L_o_ phase had a broad inhomogeneous distribution of anisotropy values, including maximum anisotropy 0.4 which indicates the dense molecular packing and low local dynamics in this phase. The average anisotropy value was 0.23. The zoom of the L_d_/L_o_ phase distribution in the ternary bilayer (Figure 10C) reveals the granular structure of local rotational mobility of the lipids.

#### 2.3.3. Combined Optical and Electrical Recording from a Horizontal Bilayer—Membrane-Integration of a Pore Forming Toxin

Apoptosis induced by pathogenic *Neisseria gonorrhoeae* depends on the bacterial porin PorB. The ATP-binding β-barrel protein is transferred to host cells during infection and targets mitochondria forming a large ion channel in the inner mitochondrial membrane. This induces breakdown of the mitochondrial membrane potential (Δ*Ψ*_m_) thus sensitizing host cells for apoptosis [52].

Using horizontal bilayer setup, we investigated by simultaneous fluorescence-optical and electrical recording the membrane insertion of PorB and its oligomeric state in solution and in the membrane. We determined the diffusion constant and the molecular brightness of PorB in the bilayer and in the surrounding buffer while simultaneously observing channel activities under controlled membrane potential. The diffusion constant was used to determine the oligomeric state of electrically active PorB in the bilayer.

**Figure 11:**
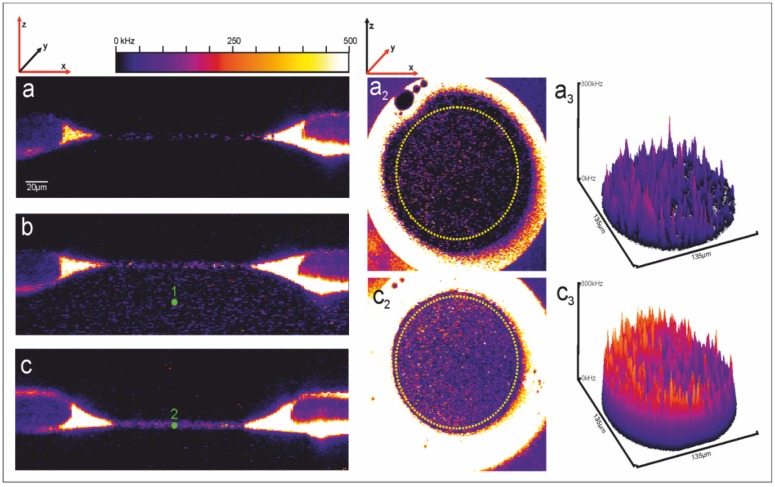
Horizontal bilayer laser scanning fluorescence recordings with PorB in solution and in the membrane.

Figure 11a shows a laser-scanning graph of a horizontal bilayer in the *xz* plane. Only background fluorescence is observed from the azolectin type IV-S lipid membrane. After addition of 1 nM PorB-637 to the *trans*-compartment (b) the porin accumulated in the membrane. Excess PorB-637 was washed out using perfusion (c). The accumulation of PorB-637 in the bilayer is quantified in (a_3_) and (c_3_).

**Figure 12:**
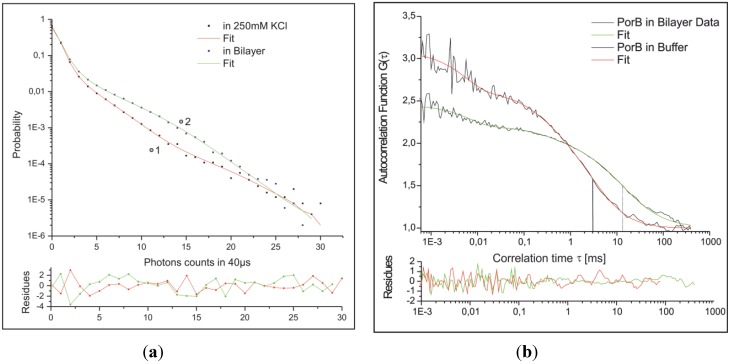
(**a**) FIDA and (**b**) FCS analysis of recorded fluorescence fluctuations (*c*_buff_ = 0.3 nM, *Q*_buff_ = 40 kHz, *c*_mem_ = 0.2 nM, *Q*_mem_ = 88 kHz, *D*_buff_ = 31 µm^2^/s, *D*_mem_ = 4.1 µm^2^/s).

Fluorescence fluctuations were recorded directly after addition of 1 nM (final concentration) labeled PorB-637 to the *trans*-compartment (see flag “1” in Figure 11b and Figure 15 for labeling procedure) and subsequently after perfusion (20 times of the total trans-compartment volume) directly in the bilayer (flag “2” in Figure 11c). Data were analyzed by FCS and FIDA. Figure 12a shows the fluorescence intensity distribution (FIDA) traces with the corresponding fit which calculates the concentration and the brightness of PorB-637 in the buffer (Figure 11b) and the bilayer (Figure 11c), respectively. The changes in the molecular brightness are likely to be attributed to changes of the dielectrical environment of the fluorophore upon integration of PorB into the membrane for the following reasons: PorB forms a stable trimer in detergent-buffer solutions [52,53] and does not change its oligomeric state upon functional incorporation into the membrane [52]. Therefore oligomerization of the protein cannot explain the increase in the molecular brightness of the fluorophore upon integration of the labeled protein. Atto637 is a zwitterionic dye which can be sensitive to membrane surface properties like dielectric constants and surface pH [54]. When we used the Atto532-NHS dye for labeling of PorB, no changes in the brightness of the dye were observed after incorporation of the labeled protein into the membrane (data not shown). Fluorescence correlation analysis was used to determine the diffusion coefficient of PorB-637 in solution and in the bilayer. Figure 12b shows the FCS curves and the corresponding fit for measurement of PorB in solution (flag “1” in Figure 11b) and PorB in the membrane (flag “2” in Figure 11c). The autocorrelation data from the measurement in solution and in the membrane were fitted with a 3D-2-component-triplett-model according to Meseth *et al.* (1999) [55]. The obtained diffusion times and amplitudes of the components for PorB in solution were: $\tau_{1}=276\;\mu s\;\left(29\%\right)$ and $\tau_{2}=3.35\;\text{ms}\;\left(71\%\right)$. These diffusion times and corresponding amplitudes indicate that most of the labeled PorB was contained in larger detergent micelles. For a homogeneous spherical trimeric PorB with the known dimensions [53], a diffusion time of $\tau_{PorB}^{trimer}=1.1.\;\text{ms}$ using the known diffusion Einstein-Stokes equation $\left(D=kT/(6\pi \eta r_{PorB})\right)$ would have been expected in buffer solution. The obtained diffusion times and amplitudes of the components for PorB in/at the membrane were: $\tau_{1}=500\;\mu s\;\left(21\%\right)$ and $\tau_{2}=14.67\text{ms}\;\left(79\%\right).\;$ These diffusion times and corresponding amplitudes indicate that most of the labeled PorB was incorporated into the membrane. We propose that the slow diffusing fraction ($X_{s}=0.79$) with *D* = 4.1 µm^2^/s represent the PorB fraction in the membrane. Using the known dimensions of the PorB [53], we tested this proposal by comparing the diffusion of PorB within the membrane with the one of a model peptide (L18). L18 is a hydrophobic poly-leucine model-peptide (AKK-(L_18_)-GKK-Atto637) with a radius of 5.5 Å capped at both ends by polar heads which forms a stable and well-defined α-helix as model of a transmembrane cylinder with a height of 28 Å [56].

**Figure 13:**
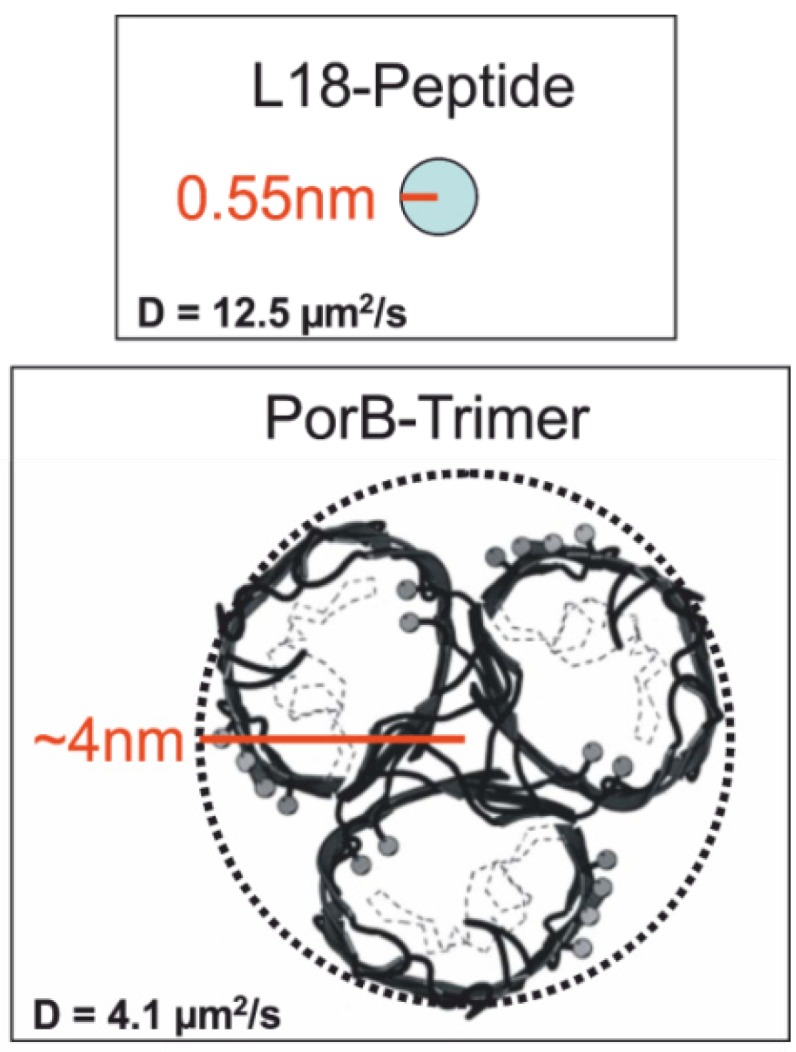
Size of the electrically active PorB.

The diffusion coefficient of the PorB-637 membrane fraction *D* = 4.1 µm^2^/s and its known size (Figure 13) was used to determine the apparent membrane viscosity by the Saffman–Dellbrück fit (Equation 4).

(4) $$D_{lat}=\frac{kT}{4\pi \eta h}\;\left(\text{ln}\frac{\eta h}{\mu_{w}a}-\gamma \right)$$

The lateral diffusion coefficient $D_{lat}$ is dependent on the surface viscosity η, the membrane thickness h=2.8 nm (L18),  h=5 nm (PorB), the viscosity of the surrounding aqueous medium $\mu_{W}=1\;\text{mPa}∙\text{s}$ and the radius of the diffusing particle *a*. *γ* is the Euler constant. For PorB membrane diffusion we obtained an apparent membrane viscosity of $\eta_{mem}=0.058\;\text{Pa}∙\text{s}$. Accordingly, for the transmembrane spanning peptide L18 with *D* = 12.5 µm^2^/s (Figure 13), we obtained an apparent membrane viscosity of $\eta_{mem}=0.045\;\text{Pa}∙\text{s}$ a value which compares well with the one obtained from PorB membrane diffusion. We therefore conclude that the slow-diffusing fraction of PorB represents the membrane inserted PorB.

#### 2.3.4. Electrical Recording from the PorB Containing Horizontal Bilayer

Current recordings were performed in the voltage clamp configuration as described in detail elsewhere [19].

**Figure 14:**
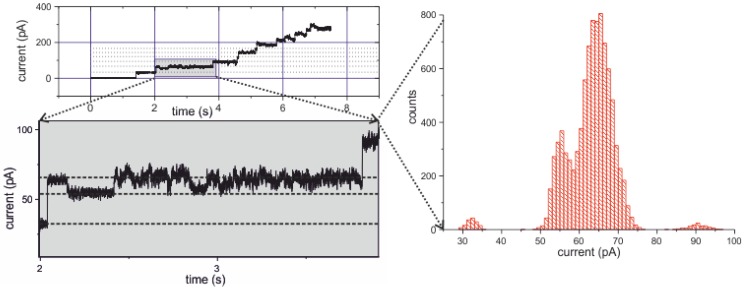
Current recordings from the bilayer shown in Figure 11a.

Figure 14 shows the current trace from a bilayer with an applied *V*_m_ = 10 mV. Rapidly after addition of PorB-637 to the *trans*-compartment (Figure 11b), multiple channel incorporations were detected electrically. The histogram of the expanded view reveals a conductance value for the trimer single channel currents in the same order of magnitude as observed previously [52].We have previously shown that the single active unit of PorB is formed by trimer containing three channel pores which are gated independently. Beside the main and maximal open state of a single channel of $G_{main}^{max}=420\;\text{pS}$, smaller subconductance states were observed. Importantly, PorB inserts always as trimer into the membrane with all three pores being in the open state at membrane potentials below 60 mV [52]. Thus, insertion events as shown in Figure 14 with a conductance increment of $\text{Δ}G\tilde{=}1\;\text{pS}$ correspond to a trimeric PorB unit. Moreover, when the membrane voltage was increased to values above 100 mV, nearly all channels closed as observed previously with the unlabeled PorB (details not shown).

Interestingly, when comparing the number of PorB trimers inserted into the membrane as obtained from the FCS measurements (Figure 11c and Figure 12) with the number of active channels obtained from the electrical recording (Figure 14) we have to recognize that only 4.5% of the inserted PorB trimers were active (see Appendix).

## 3. Experimental Section

### 3.1. Chemicals

l-α-phosphatidylcholine type IV-S ≥30%, Cholesterol (CO), Distereoyl-*sn*-glycero-3-phosphocholine (DSPC), Poly(l-lysine)-g-poly(ethyleneglycol) (PLL-g-PEG) and *n*-decane (ReagentPlus, ≥99%) were purchased from Sigma-Aldrich (St. Louis, MO, USA). 2-Dioleoyl-*sn*-glycero-3phosphocholine (DOPC), 1,2-dimyristoyl-*sn*-glycero-3-phosphocholine (DMPC) were purchased from Avanti Polar Lipids Inc. (Alabaster, AL, USA). Atto647N-1,2-dihexdecanoyl-*sn*-glycero-3-phosphatidyl-ethanolamine (DPPE647N) was purchased from Atto-Tec GmbH (Siegen, Germany). Lipids were stored in methanol/chloroform (1:1) at −20 °C under nitrogen. Agarose was purchased from Carl Roth GmbH+Co. KG (Karlsruhe, Germany). The PTFE foil (Chemfilm^®^ DF1000) was obtained from Saint-Gobain Performance Plastics Corporation (Hoosick Falls, NY, USA). Type-F low auto-fluorescence immersion oil was purchased from Olympus Corporation (Tokyo, Japan). All the rest of the reagents and solvents were obtained from Carl Roth GmbH+Co. KG (Karlsruhe, Germany), Applichem GmbH (Darmstadt, Germany), Biomol GmbH (Hamburg, Germany) or Sigma-Aldrich (St. Louis, MO, USA) and were of analytical grade.

### 3.2. Imaging

FCS measurements were performed on an Insight Cell 3D microscope connected to avalanche photodiode detectors (SPCM-AQR-13-FC) from Perkin Elmer Optoelectronics (Fremont, CA, USA). The instrument is equipped with a 40× water immersion objective (UApo340, NA 1.15) from Olympus Corporation (Tokyo, Japan), a 488 nm argon-ion laser from JDS Uniphase Corporation (Milpitas, CA, USA), a 543 nm continuous wave HeNe laser, a 635 nm pulsed diode laser both from PicoQuant (Berlin, Germany). Signals are split up according to polarization and wavelength on the correlator and the imaging unit and on a PHR 800 router connected to a PicoHarp 300 counting module both from PicoQuant, as well (for details see [28]).

Tracking of single molecules was performed on a wide-field system based on a modified OLYMPUS IX81 microscope (Olympus Europa Holding GmbH, Hamburg, Germany). A custom-built arc-lamp-laser-switch provides an additional sideport for the laser illumination between the standard fluorescence illuminator IX2-RFA (Olympus) and the hg-lamp. A red HeNe Laser (LSRP-3001, 632.8 nm, 30 mW, Research Electro Optics, Boulder, Colorado 80301) is coupled via fiber optics (Laser Beam Coupler 60SMS-1-4-A6.2S-02, Polarization-maintaining singlemode fiber cable PMC-630-4,3-NA012-3-APC-200-P, Fiber Collimator 60FC-T-4-M20-04, Schäfter + Kirchhoff GmbH, D-22525 Hamburg, Germany ) in a micro-bench-element (Qioptiq Photonics GmbH and Co KG (Formerly LINOS), Göttingen, Germany) which provides flexibility if further rayforming is needed. ND-filters (Qioptiq) were placed in the microbench. The microbench is coupled via two adjustable dichroitic mirrors (Qioptiq) to the switch. The emerging beam from the fiber collimator has a diameter (1/e^2) of 4 mm and is focused by the IX2-RFA-condensor (Olympus) which has a focal length of about 210 mm (measured in an optical bench and calculated via the abbe-method) in the backfocal plane of the objective. Resulting together with the 60x objective (UPLSAPO 60XO, NA 1.35, f = 3 mm, Olympus) in a spot diameter of 57 µm (1/e^2). Finetuning of the spotsize was possible via slight defocusing of the collimator or simply with the adjustable field stop of the IX2-RFA. The max. power at the object-plane was 9 mW (450 W/cm2, ~0.25 Exc-Photons/µs/absorption cross-section). The filters were a DC beamsplitter (LPD01-633RS, RazorEdge 633 RS) and a blocking filter (LP02-633RS, RazorEdge LP 633 RS, both Semrock, Rochester, New York, USA/AHF, Tübingen, Germany). For image/video-capturing an EMCCD (ImagEM C9100-13 EM-CCD, Hamamatsu, Japan) was connected to the left sideport of the IX81.

### 3.3. The Electro-Mechanical Micro-Hole Generator (EMMHG)

The electro-mechanical micro-hole generator (EMMHG) has three main components: (i) A central microchip-/Teflon sheet-assembly where the Teflon foil is fixed, (ii) a micro-needle holder with a custom-built micro-stepper device combined with custom build microscope and (iii) a spark-gap based on micro-needles with adjustable distance. An electronic control unit driving the ignition coil allows for the generation of single high-power pulses with variable power.

One of the side tungsten needles (tip diameter ~1–5 µm) allows optical controlled coarse and fine tuning in both lateral (*x*/*y*) directions, related to the optical axis (*z*) of the microscope. The used combination of objective-lens allows resolving holes down to 5–10 µm in diameter. After generating the small hole (~5 µm) in the foil, it is placed in the spark-gap-unit between the two aligned electrodes, each placed in the same lateral control unit as the needle. HV-generated spark-arches were then used to smooth the surfaces of the micro-hole.

### 3.4. Bilayers

The horizontal bilayer chip is self-built from polytetrafluoroethylene (PTFE) since this material provides the required dielectric strength and chemical persistence. By use of double-sided adhesive tape a perforated PTFE foil (aperture diameter ≤100 µm) is placed on the chip. Another adhesive tape carrying a notch which represents the *trans* channel is stacked on the PTFE foil and the chip is sealed by a cover slip (see Figure 1A). The distance between cover slip and PTFE foil amounts to 100 µm and lies well in the working range of a high numerical aperture objective.

For agarose supported bilayers the adhesive tape containing the *trans* channel is placed on the cover slip in the first place. Then the segment where the PTFE foil of the *cis* compartment will be located is covered by a non-sticky foil and hot agarose is filled underneath. After polymerization of the agarose, the cover is removed and the remaining parts of the bilayer chip are assembled as described above.

For PLL-g-PEG supported bilayers the PLL-g-PEG (1 mg/mL in 250 mM KCl, 20 mM MOPS/Tris, pH 7.2) is placed onto a piranha etch-cleaned, air-dried cover slip, and was left to attach to the glass surface for 30 min. The PTFE foil was cut into shape to almost fit the diameter of the *cis* compartment and the *trans* channel was enlarged to avoid contact to the PTFE foil. By doing this, both double-sided adhesive tapes stuck together, thereby reducing the distance between cover slip and PTFE foil compared to the original assembly. This made it possible to use hydrostatic pressure to press the bilayer onto the PLL-g-PEG support.

Bilayers for freestanding, agarose and PLL-g-PEG supported measurements were formed by the panting technique. Therefore lipid stock solutions (stored in 1:1 methanol/chloroform) were mixed as required and dried under vacuum, re-solubilized in *n*-decane to a final concentration of 50–60 mg/mL and painted on the aperture using a bended, blunt-ended micro syringe.

For glass-supported bilayers DOPC and DPPE647N were mixed at a molar ratio of 1:1.3 × 10^9^, dried under vacuum and re-solubilized with buffer (250 mM KCl, 10 mM MOPS/Tris, pH 7.2) to yield lipid vesicles at a final concentration of 5 mg/mL. Sonification was used to prepare small SUVs from the vesicles. The measurements were performed in a chamber consisting of a greased rubber band that was placed onto a piranha etch-cleaned, air-dried cover slip. Bilayers were made by spontaneous rupture of lipid vesicles upon contact with the glass surface. After 30 min, the chamber was washed thoroughly. To achieve long lifetime (>2–4 h) of the free-standing and agarose/PLL-g-PEG-supported bilayer, it is important to avoid electrostatic disruption by proper electrical grounding of the microchip, even if only optical recordings were performed.

### 3.5. Fluorescence Labeling of PorB

**Figure 15:**
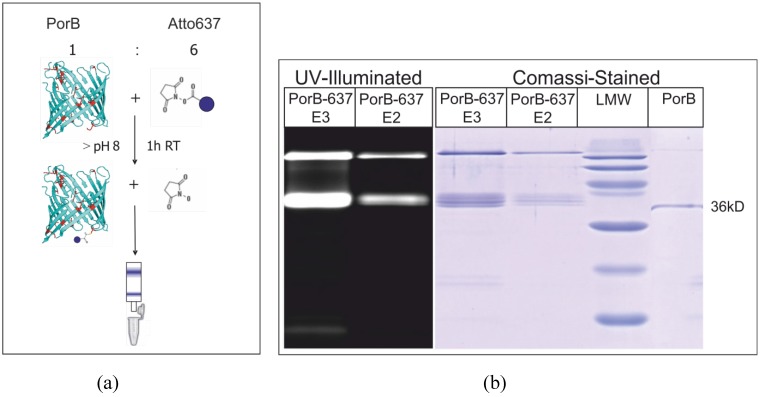
Scheme of PorB labeling (**a**), SDS-PAGE of PorB-637 verifies the labeling and purification success. Under UV-illumination fluorescent bands corresponding to PorB monomers (36 kD) and oligomers are visible (**b**).

PorB was purified as described [57]. The standard labeling protocol of the manufacturer (ATTO-TEC) was used to label lysine residues of PorB with the dye Atto637-NHS [58]. The red fluorescent dye Atto637 was covalently bound to lysine residues of PorB using an NHS-ester as a reactive group and pH 8.3 buffer. PorB (1 mg/mL = 29 µM), 0.5% Zwittergent (w/v), in 100 mM NaHCO_3_, pH 8.3, was incubated with 60 µM Atto637-NHS or 60 µM Atto532-NHS for 30 min at room temperature under stirring [58]. Excess dye was separated by size exclusion chromatography and labeling as well as purification were verified by SDS-PAGE (Figure 15b). The average degree of labeling (DOL) was determined to be ≈2, using absorption spectroscopy at 280 nm (PorB) and 637 nm (Atto637).

### 3.6. Electrical Recordings

The assembled bilayer chip is filled with buffer (250 mM KCl, 10 mM MOPS/Tris, pH 7) and agarose-covered Ag/AgCl electrodes were connected to the *cis*- and *trans*-compartment, respectively. Prepared lipid is “painted” over the aperture in the *cis*-compartment and after a stable bilayer emerged, probe was applied. Electrical recordings were performed using a CV-5-1GU headstage that was connected to a GeneClamp 500B patch-clamp amplifier. Signals were digitized by a Digidata 1322A AD/DA converter, all instruments were from Molecular Devices (Sunnyvale, CA, USA). Data was recorded using pClamp 9, also from Molecular Devices. For further readings please see [19,59].

## 4. Conclusions

The combined and simultaneous single-molecule planar bilayer electrical recording and single-molecule spectroscopy are promising approaches to probing model membrane lipid dynamics and order, as well as functional and hydrodynamic properties of integral membrane protein. Likewise, functional characteristics of ion channels or lipid-dependent mobility and distribution of the channels in the membrane can be investigated. We have described construction details, required materials and the procedure to set up a robust horizontal bilayer microchip system which optionally can be stabilized by cushions of natural or synthetic hydrogels as bilayer support. Our reports on simultaneous and combined single-molecule fluorescence–spectroscopic and electrical recordings from free standing bilayers and those which were supported by polymer cushions show that the setup can successfully be applied to examine lipid dynamics and membrane protein diffusion with simultaneous electrical single ion channel recording probing the functional state of the channel. Compared to free-floating supported planar bilayers—the described system where the bilayer spans a micro-hole, is fixed by a lipid annuls around this hole, and stabilized by a polymer cushion—the key advantage is that this bilayer still separates two water compartments with very high dielectric insulation allowing electrical check of membrane integrity. In addition, disturbance of membrane lipid dynamics and order, as well as membrane protein function, are minimized.

## Abbreviations

FCS: Fluorescence Correlation Spectroscopy
FIDA: Fluorescence Intensity Distribution Analysis
FLT: Fluorescence Lifetime

## Appendix

**Number of functional membrane inserted PorB calculated from electrophysiological measurements:**

Conductance of a single PorB monomer: $G_{monomer}=420\;\text{pS}$

Conductance of a trimeric PorB channel: $G_{trimer}=1.2\;\text{nS}$

Maximal current at V=10 mV: $I_{max}=6\;\text{nA}$

According to Ohm’s law R=U/I→G=I/U the current at V = 10 mV through a monomer and trimer at the given holding potential amounts to $I_{monomer}=4.2\;\text{pA}$ and $I_{trimer}=12.6\;\text{pA}$, respectively. Thus we can conclude that there are at least $I_{max}/I_{trimer}\;=476$ trimers incorporated in the bilayer.

**Calculations of membrane inserted PorB from the FCS measurements (see Figure 12):**

From calibration measurements with Atto655 [60] we determined the focal parameters $r_{0}=0.49\;\text{µm}$, $z_{0}=4.05\;\text{µm}$ and the axis ratio $ar=z_{0}/r_{0}=8.25$.

The fitted autocorrelation function yields $N_{bilayer}=0.832$, a fast fraction $X_{fast}=21.5\%$ with $\tau_{fast}=500\;\text{µs}$ and a slow fraction $X_{slow}=1-X_{fast}=78.5\%$ with $\tau_{slow}=14.67\;\text{ms}$.

One can now calculate the diffusion coefficients $D_{fast}={r_{0}}^{2}/\left(4∙\tau_{fast}\right)=120.294µm^{2}/s$ and $D_{slow}={r_{0}}^{2}/\left(4∙\tau_{slow}\right)=4.1µm^{2}/s$.

In order to get the absolute concentrations of the fractions we calculated the focal volume according to Schwille *et al.* [61] $V_{focus}=\sqrt{\pi^{3}}∙{r_{0}}^{2}∙z_{0}=5.42\;\text{fL}$. Hence we get a concentration of the slow fraction of $c_{slow}=\left(N_{bilayer}∙X_{slow}\right)/\left(N_{A}∙V_{focus}\right)=0.2\;nM$ with $N_{A}$ being the Avogadro constant. In line with this the concentration of the fast fraction is $c_{fast}=0.055\;\text{nM}$.

The area of the focus is $A_{focus}=\pi ∙{r_{0}}^{2}=0.756\;µm^{2}$, so the surface density of the slow fraction is $\sigma_{slow}=\left(N_{bilayer}∙X_{slow}\right)/\left(A_{focus}\right)=0.864\;µm^{-2}$. The total bilayer area can be derived from Figure 11c. With $r_{bilayer}=125/2\;µm$ the area amounts to $A_{total}=12272\;µm^{2}$. Assuming that the slow fraction represents the membrane inserted PorB then the number of inserted protein is $N_{PorB}^{inserted}=A_{total}∙\sigma_{slow}=10604$.

In relation to the number of incorporated PorB trimers from the electrophysiological measurements we can conclude that 4.49% of the inserted PorB is active.
